# Moderate consumption of fermented alcoholic beverages diminishes diet-induced non-alcoholic fatty liver disease through mechanisms involving hepatic adiponectin signaling in mice

**DOI:** 10.1007/s00394-019-01945-2

**Published:** 2019-03-16

**Authors:** Finn Jung, Tino Lippmann, Annette Brandt, Cheng Jun Jin, Anna Janina Engstler, Anja Baumann

**Affiliations:** 1 Department of Nutritional Sciences, Molecular Nutritional Science, University of Vienna, Althanstraße 14 (UZA II), 1090 Vienna, Austria; 2 Institute of Nutritional Sciences, SD Model Systems of Molecular Nutrition, Friedrich-Schiller-University Jena, Dornburger Straße 22-25, 07743 Jena, Germany; 3 Clinic for Gastroenterology, Hepatology and Infectiology, Heinrich-Heine-University Düsseldorf, Moorenstraße 5, 40225 Düsseldorf, Germany

**Keywords:** Non-alcoholic fatty liver disease, Fermented alcoholic beverages, Insulin resistance, Adiponectin

## Abstract

**Purpose:**

Results of some epidemiological studies suggest that moderate alcohol consumption may be associated with a decreased risk to develop NAFLD. Here, the effect of the consumption of moderate beer and diluted ethanol, respectively, on the development of NAFLD were assessed.

**Methods:**

Female C57BL/6J mice were fed a control diet (C-D) or a diet rich in fructose, fat and cholesterol (FFC) enriched isocalorically and isoalcoholically with beer (FFC + B) or plain ethanol (FFC + E) (2.5 g ethanol/kg body weight/day) for 7 weeks. Liver damage was assessed by histology using NAFLD activity score. Markers of inflammation, insulin resistance and adiponectin signaling were measured at mRNA and protein levels. Using J774A.1 cells as a model of Kupffer cells, the effect of alcoholic beverages on adiponectin receptor 1 (*Adipor1*) was assessed.

**Results:**

Hepatic triglyceride concentration, neutrophil granulocytes, iNOS protein concentrations and early signs of insulin resistance found in FFC-fed mice were significantly attenuated in FFC+ B-fed mice (*P* < 0.05 for all). These findings were associated with a super-induction of *Adipor1* mRNA expression (+ ~ 18-fold compared to all other groups) and a decrease of markers of lipid peroxidation in liver tissue of FFC + B-fed mice when compared to FFC-fed animals. Similar differences were not found between FFC– and FFC+ E-fed mice. Expression of *Adipor1* was also super-induced (7.5-fold) in J774A.1 cells treated with beer (equivalent to 2 mmol/L ethanol).

**Conclusions:**

These data suggest that moderate intake of fermented alcoholic beverages such as beer at least partially attenuates NAFLD development through mechanisms associated with hepatic AdipoR1 expression.

**Electronic supplementary material:**

The online version of this article (10.1007/s00394-019-01945-2) contains supplementary material, which is available to authorized users.

## Introduction

As prevalence of overweight and insulin resistance has increased markedly throughout the world in the last decades, number of individuals affected by metabolic diseases such as non-alcoholic fatty liver disease (NAFLD) has increased, too (for overview see [1]). Indeed, NAFLD, comprising a wide spectrum of liver diseases ranging from simple steatosis through steatohepatitis, fibrosis and even cirrhosis and hepatocellular carcinoma is by now the most common liver disease among the global adult population [2]. Several risk factors such as genetic predisposition, gender, age and sedentary life-style as well as nutritional intake have been identified that may influence disease development (for overview see [3, 4]). Among the latter, drinking habits and herein especially intake of alcoholic beverages are also discussed as possible critical factors of disease development (for overview see [3]); however, data are still contradictory. Indeed, results from a systematic analysis for the Global Burden of Disease Study 2017 suggest that alcohol consumption regardless of the amounts consumed bears no health benefits; however, data were not stratified according to type of beverage consumed [5]. A prospective study in the UK further showed that alcohol consumption and the presence of obesity (BMI > 30 kg/m^2^) increase the relative risk of liver disease related mortality and morbidity by a factor of 5–19 when compared to normal weight men, when 1–14 and > 15 units of ethanol per week (1 unit being equivalent to 8 g ethanol), respectively, were consumed [6]. In overweight men (BMI 25.0–29.9 kg/m^2^) these associations were also present; however, less pronounced [6]. Contrasting these findings, Dunn et al. 2012 and Moriya et al. 2011 reported that alcohol consumption at moderate amounts (< 20 g ethanol per day for men; < 10 g ethanol per day for women) is associated with a decreased prevalence of steatohepatitis in NAFLD patients [7, 8]. Furthermore, in a recently published study it was shown that light to moderate alcohol intake (light drinkers consuming < 20 g/day and moderate drinkers ingesting > 20 g/day for any period) in diabetic patients with NAFLD was not associated with liver fibrosis [9]. Reasons for these contradictory findings have not yet been fully understood; however, so far, in most of the studies participants were not stratified according to their consumption of fermented and distilled alcoholic drinks. Indeed, results of studies in patients suffering from liver cirrhosis consuming alcohol but also animal studies using high concentrations of alcohol (6.5 g EtOH/kg/bw) suggest that the kind of alcoholic drink might be critically affecting the progression of liver disease [10–12]. For instance, it has been shown that secondary plant compounds such as xanthohumol and iso-α-acids found in hop or resveratrol found in grapes at least in experimental settings may beneficially affect the development of liver diseases of various etiologies including NAFLD [13–15]. Whether these or other compounds found in fermented but not distilled alcoholic drinks are at least in part involved in the conflicting results of epidemiological studies has not yet been clarified.

Starting from this background, using a mouse model, the primary focus of the present study was to assess whether the effects of moderate consumption of a fermented alcoholic drink such as beer on the development of diet-induced NAFLD and insulin resistance differ from those of diluted ethanol and if so to determine molecular mechanisms underlying these differences.

## Methods

### Animals and treatment

All procedures were approved by the local Institutional Animal Care and Use Committee (Jena, 02–019/14) and were carried out with female C57BL/6J mice (Janvier SAS) as it was shown before that female mice are more susceptible to both, fructose-induced NAFLD and alcohol-induced liver damage when compared to male mice [16, 17]. Animals were housed in a specific-pathogen-free barrier facility accredited by the Association for Assessment and Accreditation of Laboratory Animal Care. After an adaptation to the liquid control diet (15.7 MJ/kg diet: 69% of energy (E%) from carbohydrates, 12 E% fat, 19 E% protein, Ssniff^®^, Germany) for 7 days, 48 mice were randomly assigned to the following 6 different feeding groups (8 mice per group): control diet (C-D); control diet + ethanol (C-D + E); control diet + beer (C-D + B); fructose-, fat- and cholesterol-rich diet (FFC); fructose-, fat- and cholesterol-diet + ethanol (FFC + E) and fructose-, fat- and cholesterol-rich diet + beer (FFC + B). Caloric content and macronutrient composition of the FFC, which was obtained from Ssniff^®^, Germany was as follows: 17.8 MJ/kg diet: 60 E% carbohydrates, 25 E% fat, 15 E% protein with 50% wt/wt fructose and 0.16% wt/wt cholesterol. Further details of the composition of the two diets are summarized in Online Resource 2. Animals were pair-fed the different diets for 7 weeks as detailed previously [18]. In brief, in the four groups fed ethanol or beer, the liquid diet was iso-alcoholically enriched with either pilsner beer (alcohol by volume: 4.9%) or plain ethanol (2.5 g EtOH/kg body weight) (VWR International). To ensure equal caloric intake, mean daily caloric intake of each group was assessed. The group with the lowest intake had ad libitum access to their respective diet whereas all other groups received the amount of diet consumed by the ad libitum group the day before. Body weight of each mouse was assessed weekly. Beer or plain ethanol additions to the diets were adjusted daily in accordance with dietary intake and development of body weight to ensure sufficient and equal ethanol uptake. The mice had free access to tap water throughout the whole experiment. In the 5th week of feeding a glucose tolerance test (GTT) was performed as detailed previously [19]. In brief, the mice were fastened for 6 h and narcotized before a glucose solution was injected (2 g/kg body weight; i.p.). Blood glucose levels were measured by blood sampling from tail vein at 15, 30, 60, 90 and 120 min with a standard glucometer (Bayer Vital GmbH, Germany) after glucose injection. In week seven mice were killed after being anesthetized with a mixture of ketamine and xylazine (100 mg/kg body weight ketamine, 16 mg/kg body weight xylazine, Sigma Aldrich Chemie GmbH, Germany) by cervical dislocation (see Online Resource 1 for an overview of the study design). Blood samples were taken from the portal vein just prior to killing and liver and adipose tissue were fixed in 4% neutral buffered formalin, immediately deep frozen at − 80 °C.

### Cell culture

J774A.1 cells (DSMZ, Germany) were cultured at 37 °C in a humidified, 5% carbon dioxide atmosphere with Dulbecco Modified Eagle Medium (DMEM; Pan Biotech, Germany) enriched with 1% penicillin–streptomycin (Pan Biotech, Germany) and 10% fetal bovine serum (Pan-Biotech). At 90% confluence cells were incubated with DMEM containing 2 mmol/L plain ethanol (alcohol by volume: 99.9%, VWR International) or beer for 2 and 6 h in a modified alcohol vapor chamber as described by others [20]. Cells were then harvested with peqGOLD TriFast (VWR, Germany) and stored at − 80 °C for subsequent RNA isolation.

### Parameters of liver damage

Hepatic lipid accumulation was measured as previously described [21]. In brief, triglycerides were extracted using the method of Folch et al. [22]. Triglyceride concentrations were then determined by spectrophotometry using a commercially available kit (Randox, UK). Paraffin-embedded liver samples were cut into 4 µm sections, stained with hematoxylin and eosin (H&E) and NAFLD activity score (NAS) was evaluated (Leica DM 6B, Leica, Wetzlar, Germany) according to Kleiner et al. [23]. Alanine aminotransferase (ALT) and aspartate aminotransferase (AST) activities were measured in heparinized plasma in the routine laboratory at the University Hospital of Jena (Architect^®^, Abbott, Germany).

### Plasminogen activator inhibitor 1 (PAI-1) ELISA

Deep frozen liver tissue was homogenized in TBS buffer (TRIS base: 40 mmol/L, NaCl: 140 mmol/L) and PAI-1 protein concentration was measured using a commercially available ELISA kit following the manufacturer instructions (Loxo GmbH, Germany).

### RNA isolation and real time RT-PCR

As results of other groups suggest that ethanol intake can modulate adiponectin expression in visceral adipose tissue and thereby at least in part affect the development of liver disease [24, 25], expression of genes involved in the regulation of adiponectin were determined in visceral adipose tissue and liver. Therefore, total RNA was isolated as previously described [26] and cDNA was synthesized with a commercial available kit (Promega GmbH, Germany). Integrity of RNA was assessed using gel electrophoresis and only samples without the signs of degradation were used for further measurements (liver tissue: *n* = 7–8, adipose tissue: *n* = 4–6). PCR mix was prepared using iTaq™ Universal SYBR^®^ Green Supermix (Bio-Rad Ges.m.b.H., Austria). Reactions were carried out with a CFX Connect™ Real-Time PCR Detection System (Bio-Rad Ges.m.b.H, Austria). Primer sequences used for RT-PCR are listed in Online Resource 3.

### Immunohistochemical staining of inducible nitric oxide synthase, 4-hydroxynonenal protein adducts and 3-nitrotyrosine protein adducts in liver tissue

Paraffin-embedded liver samples were cut into 4 µm sections and stained to detect inducible nitric oxide synthase (iNOS), 4-hydroxynonenal protein adducts (4-HNE) and 3-nitrotyrosine protein adducts (3-NT) using monoclonal (3-NT: Santa Cruz, USA) and polyclonal (4-HNE: AG Scientific, USA, iNOS: Affinity BioReagents, USA) antibodies. To detect specific binding of primary antibodies, tissue sections were incubated with a peroxidase linked secondary antibody and diaminobenzidine (Peroxidase Envision Kit; Dako, Hamburg, Germany). Using an image acquisition and analysis system incorporated in the microscope, the extent of staining in liver sections was defined as percent of the field area within the default color range determined by the software. To determine means, data from eight fields of each tissue section (200× magnification) were used.

### Statistical analyses

Data are presented as means ± standard error of the means (SEMs). Grubb’s test was performed before statistical analysis to identify outliers (GraphPad Prism Software, USA). In cases of unequal variances raw data were logarithmically transformed. Homogeneity of variance was tested using Bartlett’s test. One-way ANOVA with Tukey’s post-hoc test was used to determine statistically significant differences between treatment groups (GraphPad Prism Software, USA). *P* ≤ 0.05 was selected as the level of significance, NS, *P* > 0.05.

## Results

### Effect of moderate alcohol and beer consumption on body weight and liver status

**Fig. 1:**
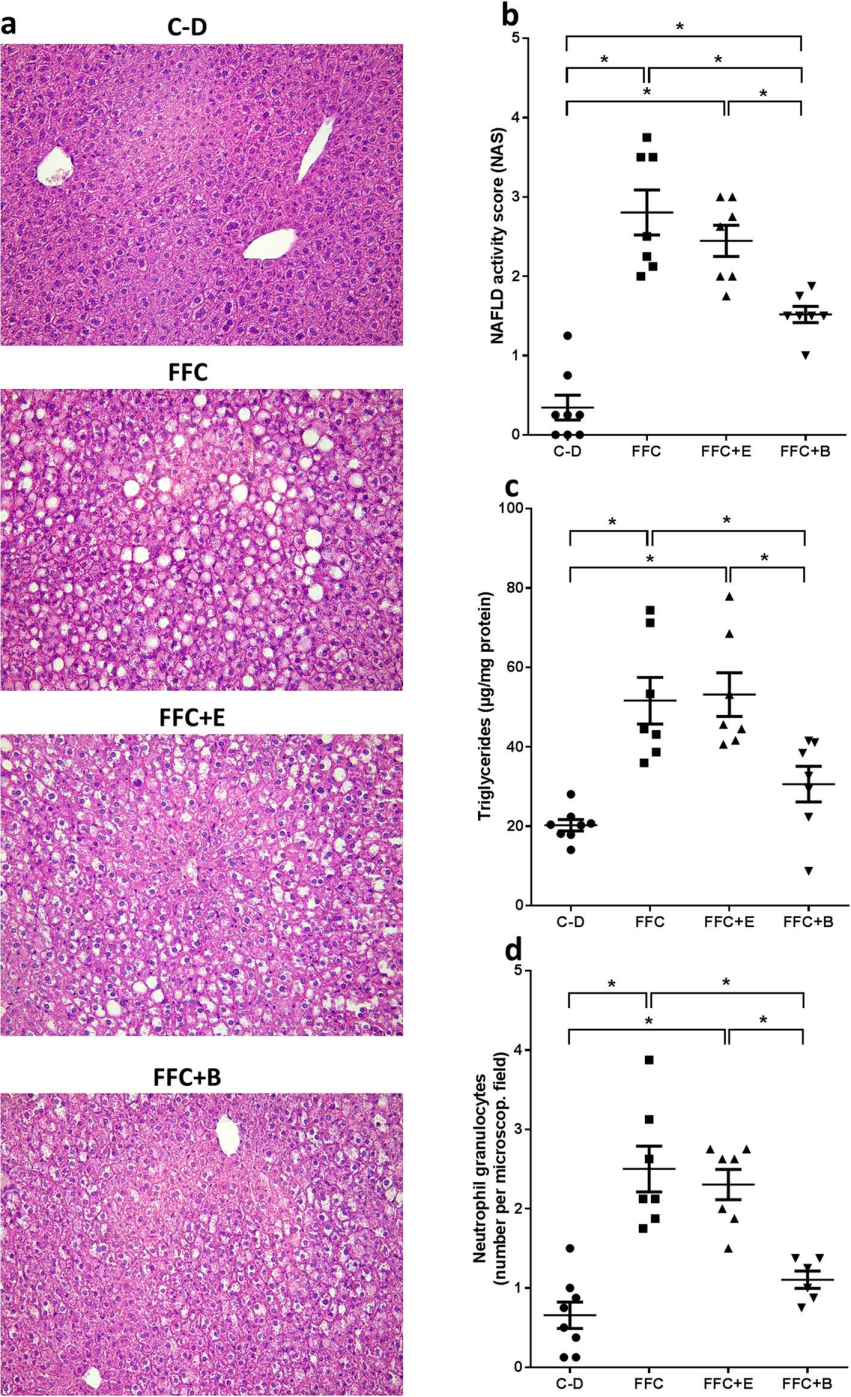
Indices of liver damage in mice fed a C-D or FFC diet isocalorically enriched with ethanol or beer for 7 weeks. **a** Representative pictures of hematoxylin and eosin staining of liver sections (200×), **b** evaluation of liver histology using NAS [23], **c** hepatic triglyceride concentrations and **d** number of neutrophil granulocytes in liver tissue. Values are means ± SEMs, *n* = 7–8. *= *P* ≤ 0.05, NS, *P* > 0.05. *B* beer, *C-D* control diet, *E* ethanol, *FFC* fructose-, fat- and cholesterol-rich diet, *NAS* NAFLD activity score

Despite receiving 2.5 g EtOH/kg body weight for 7 weeks, neither controls fed beer or plain ethanol showed any signs of liver damage (e.g. fat accumulation or inflammation) as assessed by liver histology and transaminase activity in plasma or impairments of glucose tolerance (data not shown). Therefore, data from C-D are shown as representative for all three control groups. In accordance with the pair-feeding model used, body weight and absolute body weight gain were similar between groups (Table 1). However, in line with previous experiments of our group [19], isocaloric long-term intake of a FFC was associated with the development of manifest steatosis and beginning signs of inflammation in liver (Fig. 1a). Indeed, NAS, hepatic triglyceride concentration and number of neutrophil granulocytes in liver tissue were all significantly higher in FFC-fed mice when compared with C-D-fed animals (*P* ≤ 0.05) and significantly lower in FFC + B-fed mice compared to animals fed FFC and FFC + E (*P* ≤ 0.05). While these markers did not differ between FFC + E-fed mice and those only fed the FFC, hepatic triglyceride levels (*P* = 0.28) and number of neutrophil granulocytes (*P* = 0.43) were almost at the level of controls in livers of animals fed FFC + B (Fig. 1). In contrast, absolute liver weight and liver to body weight ratio were both significantly higher in all FFC-fed mice regardless of additional treatments when compared to controls (*P* ≤ 0.05) (Table 1). Activities of AST and ALT did not differ between groups (Table 1).

**Table 1:** The effect of moderate consumption of fermented and non-fermented beverages on body weight, weight gain and parameters of liver damage in mice with FFC-induced NAFLD

	Diet groups			
	C-D	FFC	FFC + E	FFC + B
Daily energy intake (kJ)	38.1 ± 0.9	37.4 ± 0.9	36.8 ± 1.0	39.7 ± 1.5
Alcohol intake (g/kg body weight)	–	–	2.5 ± 0.1	2.5 ± 0.1
Absolute body weight (g)	21.9 ± 0.5	22.3 ± 0.5	22.0 ± 0.2	22.5 ± 0.3
Absolute weight gain (g)	3.3 ± 0.3	3.5 ± 0.4	3.6 ± 0.3	3.9 ± 0.2
Liver weight (g)	1.1 ± 0.04	1.5 ± 0.03^a,b^	1.4 ± 0.03^a^	1.3 ± 0.1^a^
Liver:body weight ratio (%)	5.0 ± 0.1	6.7 ± 0.1^a,b^	6.5 ± 0.1^a,b^	5.9 ± 0.2^a^
ALT (U/L)	21.0 ± 2.0	46.0 ± 11.9	50.7 ± 9.2	21.7 ± 3.5
AST (U/L)	41.7 ± 2.4	73.7 ± 17.2	87.1 ± 15.0	43.7 ± 2.9

Values are mean ± SEMs, *n* = 7–8

*ALT* alanine aminotransferase, *AST* aspartate aminotransferase, *B* beer, *C-D* control diet, *E* ethanol, *FFC* fructose-, fat- and cholesterol-rich diet

^a^*P* ≤ 0.05 compared to C-D

^b^*P* ≤ 0.05 compared to FFC + B; NS, *P* > 0.05

### Effect of moderate alcohol and beer consumption, respectively, on fasting blood glucose levels, glucose tolerance and markers of insulin signaling in liver tissue

**Fig. 2:**
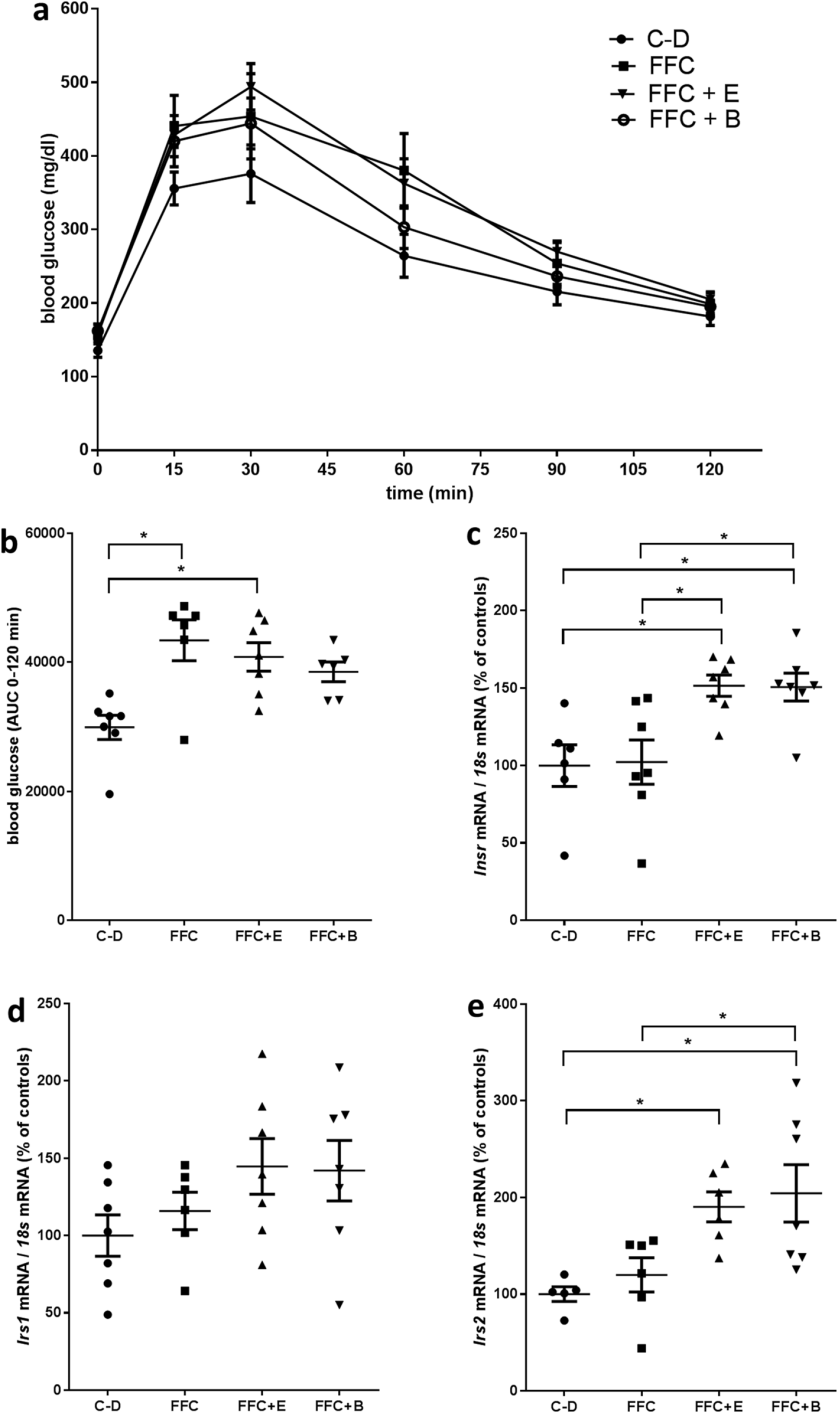
Markers of glucose metabolism and insulin signaling in C-D- and FFC-fed mice. **a** Blood glucose levels after oral administration of a glucose solution shown as **b** area under the curve. **c***Insr*, **d***Irs1* and **e***Irs2* mRNA expression normalized to 18 s mRNA. Values are means ± SEMs, *n* = 7–8. *= *P* ≤ 0.05, NS, *P* > 0.05. *AUC* area under the curve, *B* beer, *C-D* control diet, *E* ethanol, *FFC* fructose-, fat- and cholesterol-rich diet, *Insr* insulin receptor, *Irs1* insulin receptor substrate 1, *Irs2* insulin receptor substrate 2

While fasting blood glucose levels did not differ between groups, area under the curves (AUC) of the GTT of FFC- and FFC + E-fed mice were significantly higher than those of C-D-fed mice (*P* ≤ 0.05) (Fig. 2a, b). Similar differences were not found when comparing AUC of C-D- and FFC + B-fed mice (*P* = 0.07). In livers of FFC + E- and FFC + B-fed mice, expressions of insulin receptor (*Insr*) and insulin receptor substrate 2 (*Irs2*), shown to be indicative of hepatic insulin resistance [27], were significantly higher than in those of C-D-fed mice (*P* ≤ 0.05). In addition, *Insr* mRNA expression was significantly higher in livers of FFC-fed mice compared to FFC + E and FFC + B mice (*P* ≤ 0.05), whereas expression of *Irs1* mRNA did not differ between groups. Neither expression of *Insr* nor of *Irs1* or *2* in liver differed between FFC + B and FFC + E-fed mice (Fig. 2c–e).

### Effect of moderate alcohol and beer consumption, respectively, on genes involved in regulating adiponectin (*Adipoq*) expression in visceral adipose tissue and adiponectin receptor 1 and 2 (*Adipor1 and 2*) mRNA expression in liver tissue

**Fig. 3:**
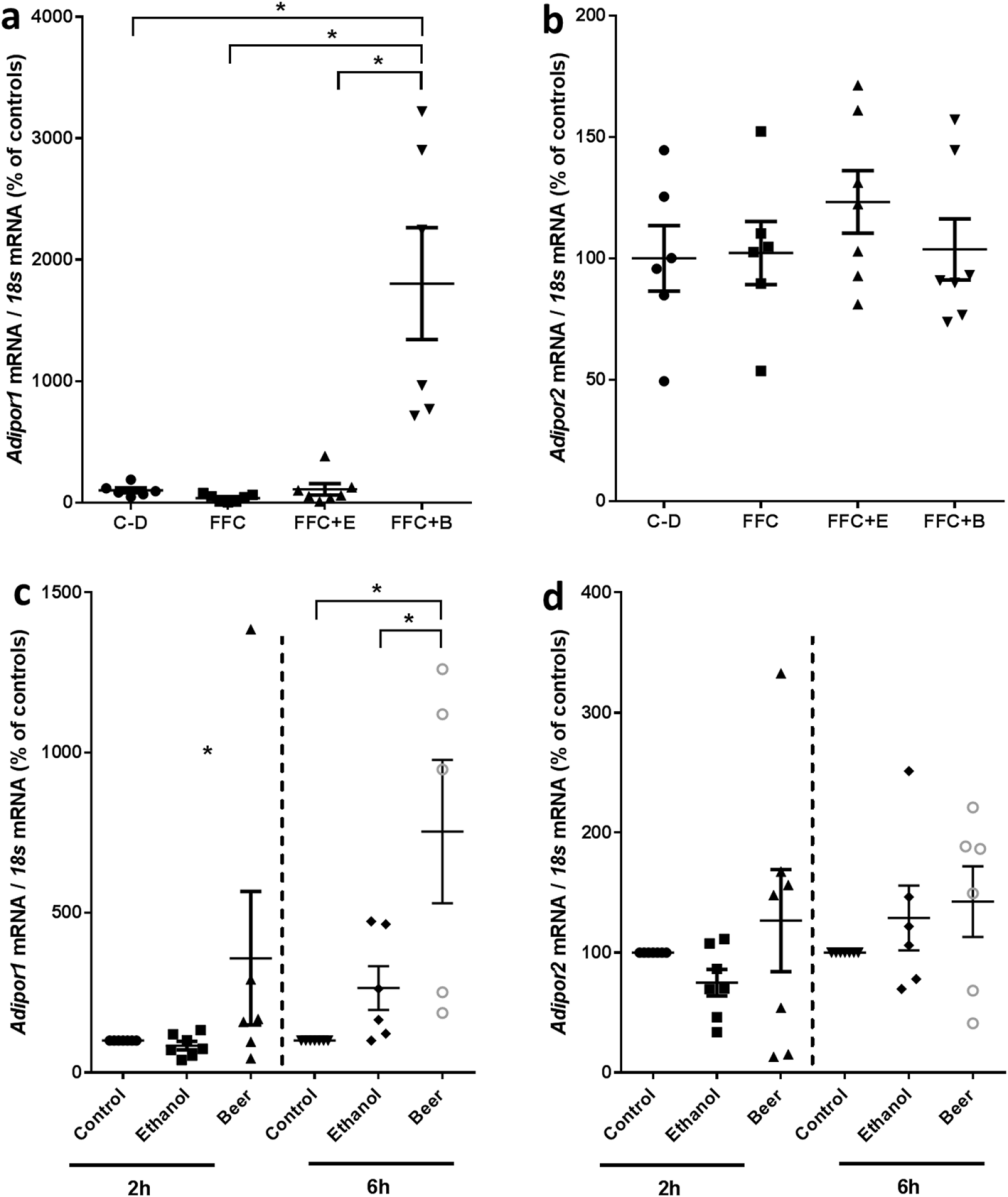
Expression of *Adipor1* and *2* mRNA in liver tissue of C-D- and FFC-fed mice and in J774A.1 cells treated with ethanol and beer for 2 and 6 h. **a***Adipor1* and **b***Adipor2* mRNA expression normalized to 18 s mRNA in liver tissue of mice (*n* = 7–8) and **c***Adipor1* and **d***Adipor2* mRNA expression in J774A.1 cells (*n* = 5–7) challenged with 2 mmol/L ethanol or isoalcoholic doses of beer for 2 and 6 h, respectively. Values are means ± SEMs. *= *P* ≤ 0.05, NS, *P* > 0.05. *Adipor* adiponectin receptor, *B* beer, *C-D* control diet, *E* ethanol, *FFC* fructose-, fat- and cholesterol-rich diet

In visceral adipose tissue expression of *Adipoq* mRNA was higher in FFC + B-fed mice when compared to all other groups; however, as data varied considerably within some groups, differences did not reach the level of significance. In line with these finding, expression of Sirtuin 1 (*Sirt1*), being critical in the regulation of *Adipoq* mRNA expression [28], was significantly higher in visceral adipose tissue of FFC + B-fed mice when compared to controls and FFC + E-fed mice (*P* ≤ 0.05). Similar differences between groups were not found for peroxisome proliferator-activated receptor 1 (*Pparγ1*) and forkhead box protein 1 (*Foxo1*) expression, respectively (Table 2). In liver tissue, expression of *Adipor1* was significantly higher in livers of FFC + B-fed mice in comparison to all other groups (+ ~ 18-fold). Similar differences were not found for *Adipor2* mRNA expression (Fig. 3a, b), which was similar between groups.

**Table 2:** Effect of moderate consumption of fermented and non-fermented beverages on markers of adiponectin production in mice with FFC-induced NAFLD

	Diet groups			
	C-D	FFC	FFC + E	FFC + B
*Pparγ1* mRNA expression (% of control)	100.0 ± 16.5	102.4 ± 30.6	99.6 ± 13.2	101.9 ± 10.4
*Foxo1* mRNA expression (% of control)	100.0 ± 26.5	143.6 ± 26.0	111.0 ± 13.6	118.3 ± 34.9
*Sirt1* mRNA expression (% of control)	100.0 ± 16.5	140.8 ± 36.8	99.6 ± 16.7	310.6 ± 98.1^a,b^
*Adipoq* mRNA expression (% of control)	100.0 ± 20.0	51.8 ± 10.8	76.6 ± 14.5	166.7 ± 47.0

Values are mean ± SEMs, *n* = 4–6

*Adipoq* adiponectin, *B* beer, *C-D* control diet, *E* ethanol, *FFC* fructose-, fat- and cholesterol-rich diet, *Foxo1* forkhead box protein O1, *Pparγ1* peroxisome proliferator-activated receptor γ1, *Sirt1* Sirtuin 1

^a^*P* ≤ 0.05 compared to C-D

^b^*P* ≤ 0.05 compared to FFC + E; NS, *P* > 0.05

### Effect of ethanol and beer on *Adipor1* and *Adipor2* mRNA expression of murine monocytes (J774A.1)

To further delineate the effects of beer on adiponectin signaling murine monocytes (J774A.1 cells), employed as a model of Kupffer cells, were incubated with a concentration of 2 mmol/L ethanol or beer for 2 and 6 h. No changes in *Adipor1* mRNA expression were found between naïve control cells and cells incubated with ethanol or beer for 2 h. In contrast, in cells incubated with beer for 6 h, *Adipor1* mRNA expression was super-induced being ~ threefold and ~ sevenfold higher than in ethanol-treated and naïve cells, respectively. In line with the findings in liver tissue of mice, expression of *Adipor2* remained unchanged throughout all treatments and time points (Fig. 3c, d).

### Effect of moderate alcohol and beer consumption, respectively, on iNOS and 4-HNE protein adduct concentration and on markers of lipogenesis in liver tissue

**Fig. 4:**
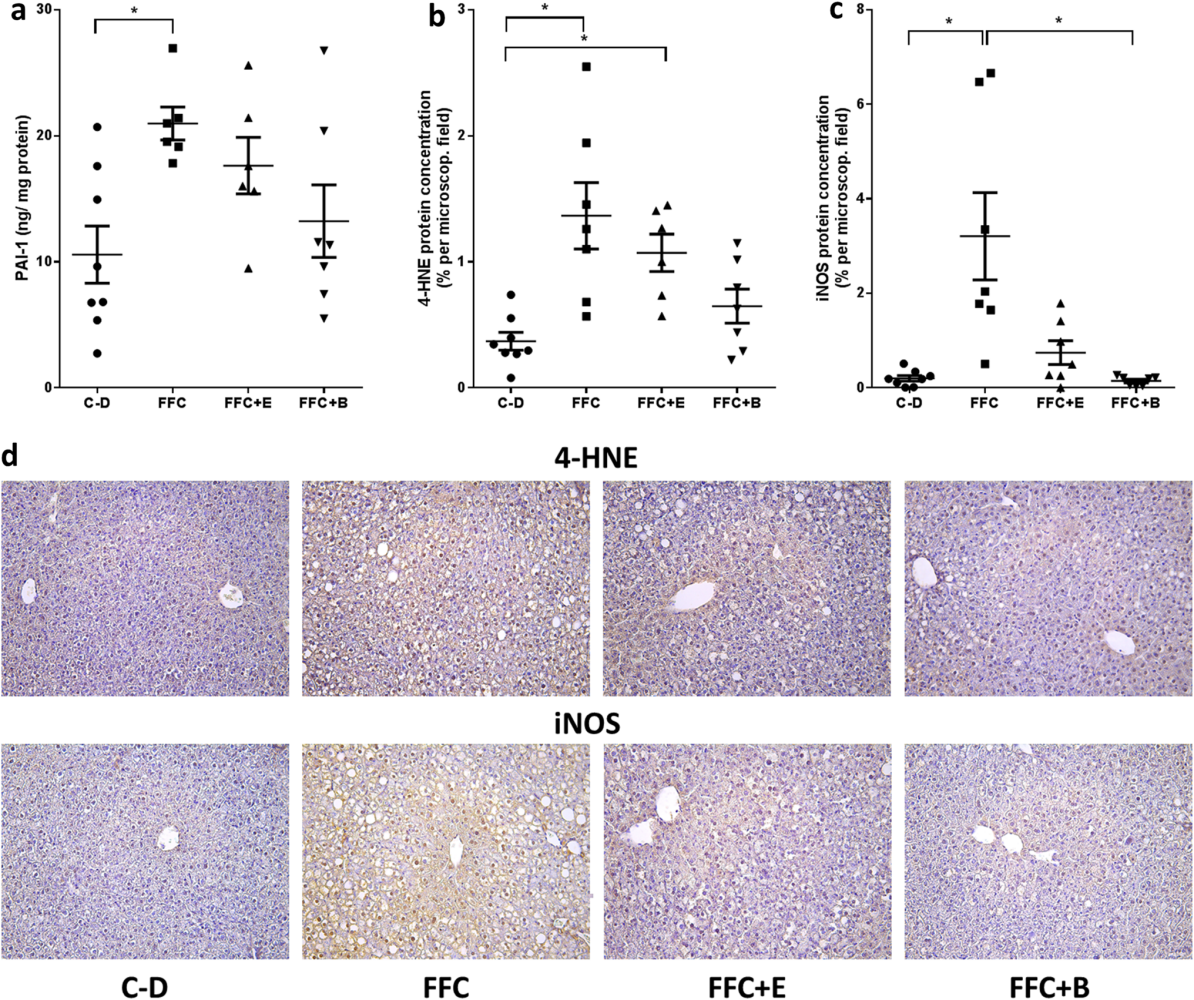
Markers of inflammation and lipid peroxidation in liver tissue of mice fed a C-D or FFC-diet enriched with beer or ethanol for 7 weeks. **a** PAI-1 protein concentrations in liver tissue, densitometric analysis of the protein staining of **b** 4-HNE and **c** iNOS with **d** representative pictures of the respective stainings. Values are means ± SEMs, *n* = 7–8. *= *P* ≤ 0.05; NS, *P* > 0.05. *4-HNE* 4-hydroxynonenal protein adducts, *B* beer, *C-D* control diet, *E* ethanol, *FFC* fructose-, fat- and cholesterol-rich diet, *iNOS* inducible nitric oxide synthase, *NAS* NAFLD activity score, *PAI-1* plasminogen activator inhibitor 1

As results of others suggest that adiponectin is critical in the regulation of inflammation and lipogenesis [29] and that especially AdipoR1 may modulate inflammation [30], markers of inflammation and lipid peroxidation were determined in livers of mice fed the different diets. Protein levels of PAI-1 were significantly higher in mice fed the FFC diet than in C-D-fed animals (*P* ≤ 0.05) while no differences were found between FFC + E- and FFC + B-fed mice and controls (Fig. 4a). Concentration of 4-HNE protein adducts was also significantly higher in FFC- and FFC + E-fed mice when compared to controls (*P* ≤ 0.05), while levels of 4-HNE protein adducts in livers of FFC + B-fed mice were almost at the level controls (Fig. 4b). In line with these findings, iNOS protein concentration in livers of FFC-fed mice was significantly higher than in livers of C-D and FFC + B-fed mice (*P* ≤ 0.05) while the latter did not differ (Fig. 4c). In addition, markers of lipogenesis were also assessed. Expressions of fatty acid synthase (*Fas*), sterol regulatory element-binding protein 1c (*Srebp-1c*) and peroxisomal acyl-coenzyme A oxidase 1 (*Acox1*) mRNA were similar between groups (Table 3).

**Table 3:** Effect of moderate consumption of fermented and non-fermented beverages on markers of lipid metabolism in mice with FFC-induced NAFLD

	Diet groups			
	C-D	FFC	FFC + E	FFC + B
*Fas* mRNA expression (% of control)	100.0 ± 24.8	132.9 ± 29.3	115.6 ± 26.2	136.0 ± 24.7
*Srebp-1c* mRNA expression (% of control)	100.0 ± 17.7	151.1 ± 18.3	171.8 ± 28.1	155.2 ± 16.8
*Acox1* mRNA expression (% of control)	100.0 ± 11.4	117.3 ± 14.2	141.4 ± 10.5	131.4 ± 5.1

Values are mean ± SEMs, *n* = 7–8. NS, *P* > 0.05

*Acox1* Acyl-Coenzyme A oxidase 1, *B* beer, *C-D* control diet, *E* ethanol, *Fas* fatty acid synthase, *FFC* fructose-, fat- and cholesterol-rich diet, *Srebp-1c* sterol regulato ry element-binding protein 1c

## Discussion

Results from some human studies suggest that moderate alcohol consumption may dampen cardiovascular and liver-associated diseases [8]; however, which molecular mechanisms are involved and whether this is an effect of ethanol as such or bound to non-alcoholic components found in some alcoholic has not yet been fully understood. Using a pair-feeding model of diet-induced NAFLD shown before to induce severe steatosis with beginning inflammation within 6–8 weeks in mice [31], we assessed the effect of moderate consumption of fermented and non-fermented beverages (2.5 g EtOH/kg body weight for 7 weeks derived from plain ethanol or beer) on diet-induced NAFLD in mice [24]. While this dose of ethanol would cause significant health impairments associated with severe signs of drunkenness in humans [32], in mice having an ~ 5 times higher ethanol elimination rate compared to humans [33], this dose of ethanol does not lead to the development of any obvious signs of liver damage (data not shown) or drunkenness. This dose of ethanol was, therefore, selected for the present study as a moderate ethanol dose for mice. Chronic intake of beer markedly attenuated the development of NAFLD as assessed by NAS, hepatic triglyceride levels and number of neutrophils while isoalcoholic doses of plain ethanol had no effects on these markers of liver damage. However, as mice did not show any obvious signs of severe liver disease and data varied considerably within groups, activities of transaminases did not differ between groups. Data from others also suggest that ALT activities are not always elevated in settings of NAFLD [34]. The results of the present study are somewhat in contrast to previous results, showing that in standard chow fed ob/ob mice similar doses of ethanol markedly attenuated the development of fatty liver disease [24]. Difference between the present study and the findings of others might have resulted from differences in study design. Indeed, while in the present study female C57BL/6J mice were fed a fructose-, fat- and cholesterol-rich diet being enriched with plain ethanol or beer to study the effects of moderate ethanol intake on liver disease, in the study of Kanuri et al. male ob/ob mice were fed standard chow and plain ethanol in drinking water [24].

The amelioration of beer on markers of NAFLD were associated with an improvement of glucose tolerance, shown to be a key risk factor in the development of NAFLD (for overview see [35]). Interestingly, while AUCs of GTT were similar between FFC- and FFC + E-fed mice and higher than in C-D- and FFC + B-fed mice, hepatic markers of insulin signaling such as *Insr* and *Irs2* were increased in livers of both FFC + E- and FFC + B-fed mice. The findings on the effects of alcohol consumption and in particular of moderate alcohol intake on insulin signaling and glucose tolerance are rather contradictory. Indeed, while in some human studies low and moderate alcohol and beer intake, respectively, were associated with impaired fasting glucose and higher risk for metabolic syndrome [36, 37], others reported that moderate intake (14–27 units/week) is associated with a reduced risk to develop type 2 diabetes with no difference between alcoholic drinks [38]. Furthermore, results of Yokoyama et al. suggest that body weight may be critical in regards to the latter findings with overweight and/or insulin resistant subjects not showing an improvement [39]. Supporting these findings from human studies, Kanuri et al. showed that in heavily overweight ob/ob mice moderate doses of ethanol had no effects on markers related to hepatic insulin signaling [24], while in the present study markers of insulin signaling in livers of normal weight FFC + E- and even more so in FFC + B-fed mice were markedly induced, which was suggested before by others [40]. The markedly better glucose tolerance found in FFC + B-fed mice might have resulted from other compounds found in beer such as hop. Indeed, results of experimental studies suggest that secondary plant compounds found in hop may improve glucose tolerance [41–43]; however, which compound or mix of compounds found in beer is critical in the beneficial effects found on glucose tolerance in the present study needs to be clarified in future studies. While the doses of beer fed to mice in the present study are within the range of a moderate alcohol consumption of a human (equivalent to 1.0 L beer in a human, considering higher ethanol elimination in mice), our finding by no means should be used to encourage anyone to drink beer to prevent the development of liver related diseases. Taken together, results of the present study suggest that in settings of diet-induced NAFLD chronic intake of moderate amounts of plain ethanol has no impact of the development of NAFLD in mice while intake of isoalcoholic and -caloric amounts of fermented beverages such as beer seem to markedly attenuate the development of this liver disease. These data by no means preclude that consumption of similar doses of ethanol or beer for a longer period of time than the one used in the present study may be associated with an abolishment of the lessening effects of beer found in the present study and rather be associated with an exacerbation of the liver disease. This needs to be assessed in future studies.

### The amelioration of moderate beer intake on the development of NAFLD are associated with a super-induction of *Adipor1* in liver tissue and a protection from lipid peroxidation

Several studies suggest that moderate and even high alcohol intake apparently irrespective of the type of alcoholic drink ingested [44] is associated with higher levels of circulating adiponectin in healthy humans [25, 45] and an induction of *Adipoq* mRNA expression in adipose tissue in rodents [24, 46]. However, results of other studies found no association or even an inverse association of alcohol consumption and circulating adiponectin levels [47–49]. Reasons for these contradictory findings have not yet been fully understood but results of Maeda et al. suggest that aldehyde dehydrogenase 2 (*ALDH2*) and alcohol dehydrogenase 1B (*ADH1B*) genotype [50] but also the availability of the redox equivalent nicotinamide adenine dinucleotide (NAD^+^), which was shown to be the limiting factor for alcohol metabolism by ADH and ALDH, might be critical [33]. Indeed, the latter studies were mainly allocated in Asia where the ALDH2/*2 genotype associated with a markedly altered ethanol metabolism is highly prevalent [51]. In support, results of in vitro studies suggest that ethanol induces *Adipoq* mRNA in an alcohol dehydrogenase-dependent manner [24]. In the present study, moderate beer but not ethanol consumption slightly induced *Adipoq* mRNA expression in adipose tissue. However, *Sirt1* mRNA expression shown to be involved in the regulation of *Adipoq* mRNA expression [28, 52] was significantly induced but neither mRNA expression of *Foxo1* nor *Pparγ1* were markedly altered in the present study in visceral adipose tissue of FFC + E- or + B-fed mice. It could be that the apparent discrepancy of the results of the present study and those of others may have resulted from the feeding model. Indeed, the diet used in the present study has been shown to induce severe impairments of glucose tolerance but also non-alcoholic steatohepatitis associated with a marked induction of proinflammatory cytokines such as tumor necrosis factor α and interleukin 6 both shown to attenuate the induction of *Adipoq* mRNA in adipocytes (for overview see [53]). It is well described that adiponectin mediates its anti-inflammatory and insulin-sensitizing effects via its respective receptors AdipoR1 and AdipoR2 (for overview see [54]). While results from knock-out experiments in mice suggest that AdipoR2-signaling affects fatty acid metabolism via AMPK and PPARα target genes, overexpression of *Adipor1* in macrophages has been shown to be associated with a reduced production of proinflammatory cytokines [30, 55]. In the present study, the less pronounced liver damage in FFC + B-fed mice was associated with a super-induction of *Adipor1* in liver tissue, while *Adipor2* expression was unchanged. *Adipor1* but not *Adipor2* was also super-induced in J774A.1 cells treated with beer whereas similar to the findings in vivo ethanol had no effects on the expression of *Adipor1* and *2*. The super-induction of *Adipor1* was associated with an attenuation of lipid peroxidation as assessed by determining 4-HNE protein adducts as well as lower iNOS and PAI-1 protein concentrations in livers of FFC + B-fed mice while in livers of FFC + E-fed mice these markers were at the level of FFC-fed animals. This is in line with findings of others investigating immunomodulatory effects of beer constituents suggesting that bitter compounds such as xanthohumol have strong anti-inflammatory, anti-oxidative and chemopreventive effects [56, 57]. If changes in *Adipor1* mRNA expression are playing a causal role in the lessening effects of beer on the development of NAFLD needs to be clarified in further studies. Furthermore, in line with the finding that *Adipor2* was unchanged throughout all groups, markers of lipogenesis were also not altered. Neither *Srebp-1c* nor *Acox1* or *Fas* differed between groups which is somewhat contrasting to previous findings employing the feeding model used in the present study [31]. The lack of induction of these markers might in part have resulted e.g. from different diet compositions.

Taken together, results of the present study suggest that moderate intake of beer may dampen the development of NAFLD and that this is associated with a super-induction of *Adipor1* in liver tissue and subsequent attenuation of inflammatory processes accompanying the disease development. Our data further suggest that immune cells such as macrophages may be critical herein. However, molecular mechanisms as well as compounds in beer involved remain to be determined.

## Conclusion

In summary, our data support findings of previous studies showing that the consumption of moderate amounts of ethanol may decelerate the development of early stages of NAFLD in rodents [24] and that this effect is even enforced when ethanol is ingested as a fermented alcoholic drink such as beer. Our data also suggest that these effects are closely related to an activation of the AdipoR1-dependent signaling cascade and subsequently a protection against lipid peroxidation and inflammation in liver tissue. Furthermore, results of the present study also indicate that compounds found in fermented alcoholic drinks such as beer even may exert health effects when consumed in rather small amounts (here: 2.5 g EtOH/kg bw/day). Since ethanol metabolism in mice is ~ 5 times higher compared to men the amount given would be equivalent to an intake of ~ 1.0 L/day in a human. Still, our findings should not encourage alcohol consumption but rather could provide a basis for further study aiming at the identification of compounds involved. Our data also by no means preclude that when consumed over an extended period of time and especially when ingested in larger doses that ethanol, be it consumed as hard liquor or beer, may lead to the development of health impairments including liver disease. Further studies are warrant to determine whether some of the `beneficial´ effects reported in previous epidemiological studies [7] are related to the consumption of fermented alcoholic beverages rather than ethanol per se as well as to identify compounds responsible for the effects found on hepatic adiponectin signaling in the present study.

## Electronic supplementary material

Below is the link to the electronic supplementary material.

Supplementary material 1 (PDF 96 KB)

Supplementary material 2 (PDF 225 KB)

Supplementary material 3 (PDF 178 KB)

## Abbreviations

3-NT: 3-Nitrotyrosine
4-HNE: 4-Hydroxynonenal protein adducts
Acox1: Acyl-coenzyme A oxidase 1
Adipoq: Adiponectin
Adipor: Adiponectin receptor
ALT: Alanine aminotransferase
AST: Aspartate aminotransferase
AUC: Area under the curve
B: Beer
C-D: Control diet
DMEM: Dulbecco Modified Eagle Medium
E%: Percentage of energy
E: Ethanol
Fas: Fatty acid synthase
FFC: Fructose-, fat- and cholesterol-rich diet
Foxo1: Forkhead box protein 1
GTT: Glucose tolerance test
H&E: Hematoxylin and eosin
iNOS: Inducible nitric oxide synthase
Insr: Insulin receptor
Irs: Insulin receptor substrate
NAFLD: Non-alcoholic fatty liver disease
NAS: NAFLD activity score
PAI-1: Plasminogen activator inhibitor 1
Pparγ: Peroxisome proliferator-activated receptor γ
SEM: Standard error of the mean
Sirt1: Sirtuin 1
Srebp-1c: Sterol regulatory element-binding protein 1c

## References

[1] Blachier M, Leleu H, Peck-Radosavljevic M, Valla DC, Roudot-Thoraval F (2013). The burden of liver disease in Europe: a review of available epidemiological data. J Hepatol.

[2] Younossi ZM, Koenig AB, Abdelatif D, Fazel Y, Henry L, Wymer M (2016). Global epidemiology of nonalcoholic fatty liver disease-Meta-analytic assessment of prevalence, incidence, and outcomes. Hepatology.

[3] Younossi Z, Anstee QM, Marietti M, Hardy T, Henry L, Eslam M, George J, Bugianesi E (2018). Global burden of NAFLD and NASH: trends, predictions, risk factors and prevention. Nat Rev Gastroenterol Hepatol.

[4] Lim HW, Bernstein DE (2018). Risk factors for the development of nonalcoholic fatty liver disease/nonalcoholic steatohepatitis, including genetics. Clin Liver Dis.

[5] Collaborators GBDRF (2018). Global, regional, and national comparative risk assessment of 84 behavioural, environmental and occupational, and metabolic risks or clusters of risks for 195 countries and territories, 1990–2017: a systematic analysis for the Global Burden of Disease Study 2017. Lancet.

[6] Hart CL, Morrison DS, Batty GD, Mitchell RJ, Davey Smith G (2010). Effect of body mass index and alcohol consumption on liver disease: analysis of data from two prospective cohort studies. BMJ.

[7] Dunn W, Sanyal AJ, Brunt EM, Unalp-Arida A, Donohue M, McCullough AJ, Schwimmer JB (2012). Modest alcohol consumption is associated with decreased prevalence of steatohepatitis in patients with non-alcoholic fatty liver disease (NAFLD). J Hepatol.

[8] Moriya A, Iwasaki Y, Ohguchi S, Kayashima E, Mitsumune T, Taniguchi H, Ikeda F, Shiratori Y, Yamamoto K (2011). Alcohol consumption appears to protect against non-alcoholic fatty liver disease. Aliment Pharmacol Ther.

[9] Patel PJ, Smith D, Connor JP, Horsfall LU, Hayward KL, Hossain F, Williams S, Johnson T, Stuart KA, Brown NN, Saad N, Clouston AD, Irvine KM, Russell AW, Valery PC, Powell EE (2017). Alcohol consumption in diabetic patients with nonalcoholic fatty liver disease. Can J Gastroenterol Hepatol.

[10] Landmann M, Wagnerberger S, Kanuri G, Ziegenhardt D, Bergheim I (2015). Beer is less harmful for the liver than plain ethanol: studies in male mice using a binge-drinking model. Alcohol Alcohol.

[11] Pelletier S, Vaucher E, Aider R, Martin S, Perney P, Balmes JL, Nalpas B (2002). Wine consumption is not associated with a decreased risk of alcoholic cirrhosis in heavy drinkers. Alcohol Alcohol.

[12] Kerr WC, Fillmore KM, Marvy P (2000). Beverage-specific alcohol consumption and cirrhosis mortality in a group of English-speaking beer-drinking countries. Addiction.

[13] Hege M, Jung F, Sellmann C, Jin C, Ziegenhardt D, Hellerbrand C, Bergheim I (2018). An iso-alpha-acid-rich extract from hops (*Humulus lupulus*) attenuates acute alcohol-induced liver steatosis in mice. Nutrition.

[14] Dorn C, Massinger S, Wuzik A, Heilmann J, Hellerbrand C (2013). Xanthohumol suppresses inflammatory response to warm ischemia-reperfusion induced liver injury. Exp Mol Pathol.

[15] Dorn C, Weiss TS, Heilmann J, Hellerbrand C (2010). Xanthohumol, a prenylated chalcone derived from hops, inhibits proliferation, migration and interleukin-8 expression of hepatocellular carcinoma cells. Int J Oncol.

[16] Spruss A, Henkel J, Kanuri G, Blank D, Puschel GP, Bischoff SC, Bergheim I (2012). Female mice are more susceptible to nonalcoholic fatty liver disease: sex-specific regulation of the hepatic AMP-activated protein kinase-plasminogen activator inhibitor 1 cascade, but not the hepatic endotoxin response. Mol Med.

[17] Wagnerberger S, Fiederlein L, Kanuri G, Stahl C, Millonig G, Mueller S, Bischoff SC, Bergheim I (2013). Sex-specific differences in the development of acute alcohol-induced liver steatosis in mice. Alcohol Alcohol.

[18] Sellmann C, Jin CJ, Engstler AJ, De Bandt JP, Bergheim I (2017). Oral citrulline supplementation protects female mice from the development of non-alcoholic fatty liver disease (NAFLD). Eur J Nutr.

[19] Sellmann C, Baumann A, Brandt A, Jin CJ, Nier A, Bergheim I (2017). Oral supplementation of glutamine attenuates the progression of nonalcoholic steatohepatitis in C57BL/6J Mice. J Nutr.

[20] Rodriguez FD, Simonsson P, Alling C (1992). A method for maintaining constant ethanol concentrations in cell culture media. Alcohol Alcohol.

[21] Kanuri G, Weber S, Volynets V, Spruss A, Bischoff SC, Bergheim I (2009). Cinnamon extract protects against acute alcohol-induced liver steatosis in mice. J Nutr.

[22] Folch J, Lees M, Sloane Stanley GH (1957). A simple method for the isolation and purification of total lipides from animal tissues. J Biol Chem.

[23] Kleiner DE, Brunt EM, Van Natta M, Behling C, Contos MJ, Cummings OW, Ferrell LD, Liu YC, Torbenson MS, Unalp-Arida A, Yeh M, McCullough AJ, Sanyal AJ, Nonalcoholic Steatohepatitis Clinical Research N (2005). Design and validation of a histological scoring system for nonalcoholic fatty liver disease. Hepatology.

[24] Kanuri G, Landmann M, Priebs J, Spruss A, Loscher M, Ziegenhardt D, Rohl C, Degen C, Bergheim I (2016). Moderate alcohol consumption diminishes the development of non-alcoholic fatty liver disease (NAFLD) in ob/ob mice. Eur J Nutr.

[25] Beulens JW, van Beers RM, Stolk RP, Schaafsma G, Hendriks HF (2006). The effect of moderate alcohol consumption on fat distribution and adipocytokines. Obesity (Silver Spring).

[26] Spruss A, Kanuri G, Stahl C, Bischoff SC, Bergheim I (2012). Metformin protects against the development of fructose-induced steatosis in mice: role of the intestinal barrier function. Lab Invest.

[27] Withers DJ, Gutierrez JS, Towery H, Burks DJ, Ren JM, Previs S, Zhang Y, Bernal D, Pons S, Shulman GI, Bonner-Weir S, White MF (1998). Disruption of IRS-2 causes type 2 diabetes in mice. Nature.

[28] Qiao L, Shao J (2006). SIRT1 regulates adiponectin gene expression through Foxo1-C/enhancer-binding protein alpha transcriptional complex. J Biol Chem.

[29] Park PH, Sanz-Garcia C, Nagy LE (2015). Adiponectin as an anti-fibrotic and anti-inflammatory adipokine in the liver. Curr Pathobiol Rep.

[30] Luo N, Chung BH, Wang X, Klein RL, Tang CK, Garvey WT, Fu Y (2013). Enhanced adiponectin actions by overexpression of adiponectin receptor 1 in macrophages. Atherosclerosis.

[31] Sellmann C, Degen C, Jin CJ, Nier A, Engstler AJ, Hasan Alkhatib D, De Bandt JP, Bergheim I (2017). Oral arginine supplementation protects female mice from the onset of non-alcoholic steatohepatitis. Amino Acids.

[32] Vonghia L, Leggio L, Ferrulli A, Bertini M, Gasbarrini G, Addolorato G, Alcoholism Treatment Study G (2008). Acute alcohol intoxication. Eur J Intern Med.

[33] Cederbaum AI (2012). Alcohol metabolism. Clin Liver Dis.

[34] Portillo Sanchez P, Bril F, Maximos M, Lomonaco R, Biernacki D, Orsak B, Subbarayan S, Webb A, Hecht J, Cusi K (2014). High prevalence of nonalcoholic fatty liver disease in patients with type 2 diabetes mellitus and normal plasma aminotransferase levels. J Clin Endocrinol Metab.

[35] Manco M (2017). Insulin Resistance and NAFLD: a dangerous liaison beyond the genetics. Children (Basel).

[36] Miyake T, Kumagi T, Hirooka M, Furukawa S, Yoshida O, Koizumi M, Yamamoto S, Watanabe T, Yamamoto Y, Tokumoto Y, Takeshita E, Abe M, Kitai K, Matsuura B, Hiasa Y (2016). Low alcohol consumption increases the risk of impaired glucose tolerance in patients with non-alcoholic fatty liver disease. J Gastroenterol.

[37] Barrio-Lopez MT, Bes-Rastrollo M, Sayon-Orea C, Garcia-Lopez M, Fernandez-Montero A, Gea A, Martinez-Gonzalez MA (2013). Different types of alcoholic beverages and incidence of metabolic syndrome and its components in a Mediterranean cohort. Clin Nutr.

[38] Marques-Vidal P, Vollenweider P, Waeber G (2015). Alcohol consumption and incidence of type 2 diabetes. Results from the CoLaus study. Nutr Metab Cardiovasc Dis.

[39] Yokoyama H (2011). Beneficial effects of ethanol consumption on insulin resistance are only applicable to subjects without obesity or insulin resistance; drinking is not necessarily a remedy for metabolic syndrome. Int J Environ Res Public Health.

[40] Paulson QX, Hong J, Holcomb VB, Nunez NP (2010). Effects of body weight and alcohol consumption on insulin sensitivity. Nutr J.

[41] Konda VR, Desai A, Darland G, Grayson N, Bland JS (2014). KDT501, a derivative from hops, normalizes glucose metabolism and body weight in rodent models of diabetes. PLoS One.

[42] Tripp ML, Darland G, Konda VR, Pacioretty LM, Chang JL, Bland JS, Babish JG (2012). Optimized mixture of hops rho iso-alpha acids-rich extract and acacia proanthocyanidins-rich extract reduces insulin resistance in 3T3-L1 adipocytes and improves glucose and insulin control in db/db mice. Nutr Res Pract.

[43] Minich DM, Lerman RH, Darland G, Babish JG, Pacioretty LM, Bland JS, Tripp ML (2010). Hop and acacia phytochemicals decreased lipotoxicity in 3T3-L1 Adipocytes, db/db mice, and individuals with metabolic syndrome. J Nutr Metab.

[44] Imhof A, Plamper I, Maier S, Trischler G, Koenig W (2009). Effect of drinking on adiponectin in healthy men and women: a randomized intervention study of water, ethanol, red wine, and beer with or without alcohol. Diabetes Care.

[45] Beulens JW, de Zoete EC, Kok FJ, Schaafsma G, Hendriks HF (2008). Effect of moderate alcohol consumption on adipokines and insulin sensitivity in lean and overweight men: a diet intervention study. Eur J Clin Nutr.

[46] Pravdova E, Macho L, Fickova M (2009). Alcohol intake modifies leptin, adiponectin and resistin serum levels and their mRNA expressions in adipose tissue of rats. Endocr Regul.

[47] Bell S, Britton A (2015). The role of alcohol consumption in regulating circulating levels of adiponectin: a prospective cohort study. J Clin Endocrinol Metab.

[48] Jung SK, Kim MK, Shin J, Choi BY (2013). A cross-sectional analysis of the relationship between daily alcohol consumption and serum adiponectin levels among adults aged 40 years or more in a rural area of Korea. Eur J Clin Nutr.

[49] Nishise Y, Saito T, Makino N, Okumoto K, Ito JI, Watanabe H, Saito K, Togashi H, Ikeda C, Kubota I, Daimon M, Kato T, Fukao A, Kawata S (2010). Relationship between alcohol consumption and serum adiponectin levels: the Takahata study—a cross-sectional study of a healthy Japanese population. J Clin Endocrinol Metab.

[50] Maeda S, Mure K, Mugitani K, Watanabe Y, Iwane M, Mohara O, Takeshita T (2014). Roles of the ALDH2 and ADH1B genotypes on the association between alcohol intake and serum adiponectin levels among Japanese male workers. Alcohol Clin Exp Res.

[51] Li H, Borinskaya S, Yoshimura K, Kal’ina N, Marusin A, Stepanov VA, Qin Z, Khaliq S, Lee MY, Yang Y, Mohyuddin A, Gurwitz D, Mehdi SQ, Rogaev E, Jin L, Yankovsky NK, Kidd JR, Kidd KK (2009). Refined geographic distribution of the oriental ALDH2*504Lys (nee 487Lys) variant. Ann Hum Genet.

[52] Iwaki M, Matsuda M, Maeda N, Funahashi T, Matsuzawa Y, Makishima M, Shimomura I (2003). Induction of adiponectin, a fat-derived antidiabetic and antiatherogenic factor, by nuclear receptors. Diabetes.

[53] Hino K, Nagata H (2012). Screening for adiponectin secretion regulators. Vitam Horm.

[54] Ghoshal K, Bhattacharyya M (2015). Adiponectin: probe of the molecular paradigm associating diabetes and obesity. World J Diabetes.

[55] Yamauchi T, Nio Y, Maki T, Kobayashi M, Takazawa T, Iwabu M, Okada-Iwabu M, Kawamoto S, Kubota N, Kubota T, Ito Y, Kamon J, Tsuchida A, Kumagai K, Kozono H, Hada Y, Ogata H, Tokuyama K, Tsunoda M, Ide T, Murakami K, Awazawa M, Takamoto I, Froguel P, Hara K, Tobe K, Nagai R, Ueki K, Kadowaki T (2007). Targeted disruption of AdipoR1 and AdipoR2 causes abrogation of adiponectin binding and metabolic actions. Nat Med.

[56] Gerhauser C (2005). Beer constituents as potential cancer chemopreventive agents. Eur J Cancer.

[57] Dorn C, Kraus B, Motyl M, Weiss TS, Gehrig M, Scholmerich J, Heilmann J, Hellerbrand C (2010). Xanthohumol, a chalcon derived from hops, inhibits hepatic inflammation and fibrosis. Mol Nutr Food Res.

