# Supplementary material for: Moderate consumption of fermented alcoholic beverages diminishes diet-induced non-alcoholic fatty liver disease through mechanisms involving hepatic adiponectin signaling in mice
Source: Eur J Nutr. 2019 Mar 16;59(2):787–99. doi: 10.1007/s00394-019-01945-2 (PMC7058579; doi:10.1007/s00394-019-01945-2)
Supplement: Supplementary file 1 — Supplementary material 1 (PDF 96 KB) [file 394_2019_1945_MOESM1_ESM.pdf]

Moderate consumption of fermented alcoholic beverages diminishes diet-induced non-alcoholic fatty liver disease through mechanisms involving hepatic adiponectin signaling in mice

Finn Jung, Tino Lippmann, Annette Brandt, Cheng Jun Jin, Anna Janina Engstler, Anja Baumann*

Article type: Original article

European Journal of Nutrition

*** Corresponding author:** Anja Baumann, Ph.D.
University of Vienna
Department of Nutritional Sciences
Molecular Nutritional Science
Althanstraße 14 (UZA II)
A-1090 Wien
Email address: anja.baumann@univie.ac.at

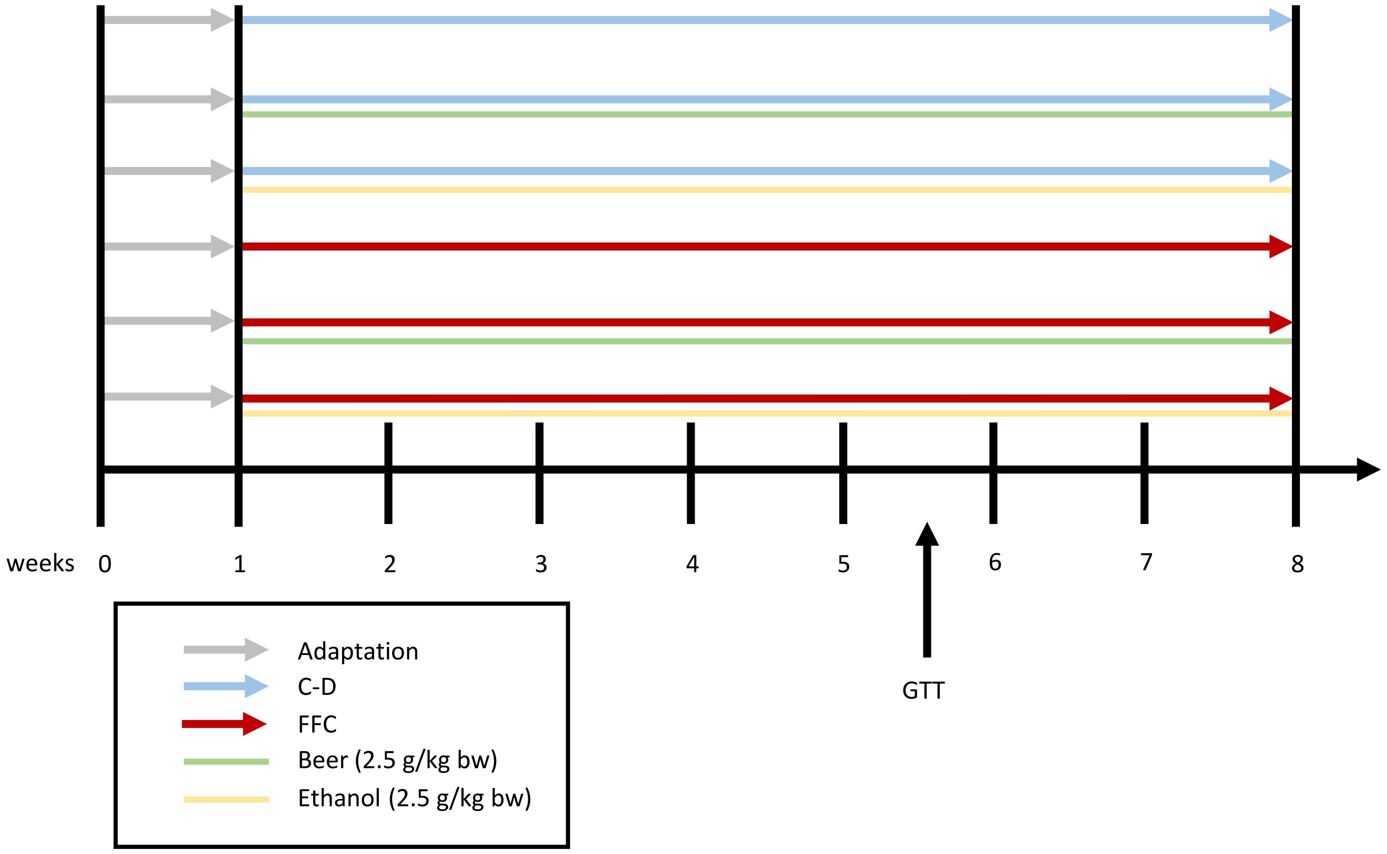

Online Resource 1: Study design. B, beer; C-D, control diet; E, ethanol; FFC, fructose-, fat-, cholesterol-rich diet; GTT, glucose tolerance test.
