# Supplementary material for: Moderate consumption of fermented alcoholic beverages diminishes diet-induced non-alcoholic fatty liver disease through mechanisms involving hepatic adiponectin signaling in mice
Source: Eur J Nutr. 2019 Mar 16;59(2):787–99. doi: 10.1007/s00394-019-01945-2 (PMC7058579; doi:10.1007/s00394-019-01945-2)
Supplement: Supplementary file 2 — Supplementary material 2 (PDF 225 KB) [file 394_2019_1945_MOESM2_ESM.pdf]

Moderate consumption of fermented alcoholic beverages diminishes diet-induced non-alcoholic fatty liver disease through mechanisms involving hepatic adiponectin signaling in mice

Finn Jung, Tino Lippmann, Annette Brandt, Cheng Jun Jin, Anna Janina Engstler, Anja Baumann*

Article type: Original article

European Journal of Nutrition

*** Corresponding author:** Anja Baumann, Ph.D.
University of Vienna
Department of Nutritional Sciences
Molecular Nutritional Science
Althanstraße 14 (UZA II)
A-1090 Wien
Email address: anja.baumann@univie.ac.at

Online Resource 2. Nutrient composition of diets.[1]

		C-D	FFC
Crude protein, CP	% wt/wt	17.4	16.0
Crude fat, CL	% wt/wt	5.1	11.8
Crude fibre, CF	% wt/wt	5.0	2.0
Crude ash	% wt/wt	4.1	4.2
Corn starch, pre-gelatinized	% wt/wt	34.6	5.0
Sucrose	% wt/wt	11.0	-
Glucose	% wt/wt	-	5.0
Fructose	% wt/wt	-	50.0
Cholesterol	% wt/wt	-	0.16
L-Lysine	% wt/wt	1.43	1.32
L-Methionine + L-Cysteine	% wt/wt	1.00	0.96
L-Threonine	% wt/wt	0.75	0.69
Calcium	% wt/wt	0.78	0.77
Phosphorus	% wt/wt	0.48	0.47
Sodium	% wt/wt	0.23	0.30
Vitamin A (retinol acetate)	IU/kg	15000	15000
Vitamin D_3 (cholecalciferol)	IU/kg	1500	1500
Vitamin E (α-tocopherol acetate)	mg/kg	150	150
Fatty acids			
4:0	% wt/wt	-	0.44
6:0	% wt/wt	-	0.29
8:0	% wt/wt	-	0.16
10:0	% wt/wt	-	0.35
12:0	% wt/wt	-	0.40
14:0	% wt/wt	0.02	1.23
16:0	% wt/wt	0.57	3.10
18:0	% wt/wt	0.18	1.14
18:1 (n-9)	% wt/wt	1.28	2.58
18:2 (n-6)	% wt/wt	2.65	0.21
18:3 (n-3)	% wt/wt	0.29	0.06
Metabolizable energy (ME)	MJ/kg	15.7	17.8
CP	kJ%	19	15
CL	kJ%	12	25
Carbohydrate, CHO	kJ%	69	60

[1] Diet modified from WSD (TD88137) and respective control diet (Ssniff®, Germany). C-D, control diet; FFC, fructose-, fat-, cholesterol-rich diet.
