# Supplementary material for: Moderate consumption of fermented alcoholic beverages diminishes diet-induced non-alcoholic fatty liver disease through mechanisms involving hepatic adiponectin signaling in mice
Source: Eur J Nutr. 2019 Mar 16;59(2):787–99. doi: 10.1007/s00394-019-01945-2 (PMC7058579; doi:10.1007/s00394-019-01945-2)
Supplement: Supplementary file 3 — Supplementary material 3 (PDF 178 KB) [file 394_2019_1945_MOESM3_ESM.pdf]

Moderate consumption of fermented alcoholic beverages diminishes diet-induced non-alcoholic fatty liver disease through mechanisms involving hepatic adiponectin signaling in mice

Finn Jung, Tino Lippmann, Annette Brandt, Cheng Jun Jin, Anna Janina Engstler, Anja Baumann*

Article type: Original article

European Journal of Nutrition

*** Corresponding author:** Anja Baumann, Ph.D.
University of Vienna
Department of Nutritional Sciences
Molecular Nutritional Science
Althanstraße 14 (UZA II)
A-1090 Wien
Email address: anja.baumann@univie.ac.at

Online Resource 3. Primer sequences used for real-time RT-PCR.

Gene	Forward 5'---3'	Reverse 5'---3'
18s	ccatccaatcggtagtagcg	gtaacccgttgaaccccatt
Acox1	gggaatttggcatcgcaga	catctccgtctgggcgtagg
Adipoq	gaagacctgcatctcctttctctcc	tcatgccgaagatgacgttactaca
Adipor1	gggacttggcttgagtggtg	gggaagaagcgctcaggaat
Adipor2	tgccctaggtaggcccaaca	tcccaaagtgcacgaccaga
Fas	tctgggccaacctcattggt	gaagctgggggtccattgtg
Foxo1	ctcccggtacttctctgctg	gtggtccagttggactggtt
Insr	catcccgaaagcgaagatcc	gagtcctgattgcatgcctgcag
Irs1	gctctagtgcttccgtgtcc	gttgccacccctagacaaaa
Irs2	gaagcggctaagtctcatgg	gacggtggtggtagaggaaa
Pparγ1	aacgtgaagcccatcgagga	ctgcacgtgctctgtgacga
Sirt1	gacgctgtggcagattgtta	gaaatcccacaggagacaga
Srebp-1c	accggctactgctggactgc	agagcaagagggtgccatcg
